# Incorporation of Digital Modulation into Vital Sign Detection and Gesture Recognition Using Multimode Radar Systems

**DOI:** 10.3390/s23187675

**Published:** 2023-09-05

**Authors:** Michael C. Brown, Changzhi Li

**Affiliations:** Department of Electrical and Computer Engineering, Texas Tech University, Lubbock, TX 79409, USA; michael.c.brown@ttu.edu

**Keywords:** multimode radar, digital radar, PMCW radar, vital-Doppler, gesture recognition

## Abstract

The incorporation of digital modulation into radar systems poses various challenges in the field of radar design, but it also offers a potential solution to the shrinking availability of low-noise operating environments as the number of radar applications increases. Additionally, digital systems have reached a point where available components and technology can support higher speeds than ever before. These advancements present new avenues for radar design, in which digitally controlled phase-modulated continuous wave (PMCW) radar systems can look to support multiple collocated radar systems with low radar-radar interference. This paper proposes a reconfigurable PMCW radar for use in vital sign detection and gesture recognition while utilizing digital carrier modulation and compares the radar responses of various modulation schemes. Binary sequences are used to introduce phase modulation to the carrier wave by use of a field programable gate array (FPGA), allowing for flexibility in the modulation speed and binary sequence. Experimental results from the radar demonstrate the differences between CW and PMCW modes when measuring the respiration rate of a human subject and in gesture detection.

## 1. Introduction

The application space of continuous wave (CW) radar systems is continuing to expand due to the small size, resolution, penetration depth, and low operation overhead of these systems. With this growth, a variety of techniques have been implemented to improve performance, gleam additional target information, and reduce interference while maintaining a low monetary and integration cost [1,2]. Of these methods, the most prominent approach that has emerged is the modulation of the carrier frequency of the radar, which allows for the range and speed of a target to be recovered. Frequency-modulated continuous wave (FMCW) radar systems are used predominantly in the automotive industry [3] and are still being explored for use in activity monitoring [4,5,6], gesture recognition [7,8,9], and vital sign detection [10,11,12,13]. In addition, improvements are still being made to CW Doppler systems used to perform vital sign monitoring and gesture detection through the in-depth analysis of processing methods [14] and gesture frame detection [15].

With the potential for radar-to-radar interference growing, investigations have been conducted into potential strategies that can reduce the spectral load values of modulated radar systems while maintaining target detection [16], the feasibility of passively measuring vital signs and gesture motion [17], and the way in which radar systems interfere with one another [18,19]. An alternative to using FMCW radar systems in ultra-wideband (UWB) radar systems has also attracted scholarly interest. These radar systems also implement frequency modulation but cover much wider bandwidths by pulsing the transmitter signal, rather than being continuous in nature. This process introduces less interference into nearby systems and can generate a higher transmission power. UWB radar systems have shown promise in performing vital sign detection [20,21], as well as short-range in-vehicle [22] and in-cabin [23] gesture detection.

In looking to expand the vital sign detection capabilities of FMCW radar systems further, multiple-input multiple-output (MIMO) radar systems have been and continue to be explored [24]. These systems take advantage of the information scaling that comes from having multiple radar systems (bistatic) [25] or a radar system with multiple coherent transmitters and receivers (monostatic) [26], thereby creating an array of virtual transmitter-receiver channels. Both MIMO variations can perform noncontact vital sign sensing and rely on orthogonal waveforms for proper operation. This orthogonality can be achieved through various methods, each with advantages and drawbacks [27]. The most common and straightforward to implement is time-division multiple access (TDMA) using alternative transmissions. This approach ensures that the transmitted waveforms do not interfere with one another as they occupy separate time windows. Digital beam forming can help improve vital sign detection [28], and alternating the transmission channels enables the full frequency bandwidth for each channel. However, this benefit comes at the cost of more time being required to build the data cube, as well as the potential loss of information as transmitters are cycled.

Another digital approach to orthogonal MIMO radar systems is through code-division multiple access (CDMA), in which the phase of each transmitter is encoded with a unique pseudorandom binary sequence (PRBS) that has high autocorrelation and low cross-correlation with other codes in the same family. By using these phase codes, all transmitters can operate at the same time as opposed to those in TDMA MIMO systems, increasing the data rate by the number of transmitters being simultaneously used. Phase codes that satisfy the orthogonality requirements of CDMA MIMO have been adapted from communication systems and include but are not limited to maximum-length sequences (MLS), Hadamard codes, Gold codes, optimal peak-to-sidelobe (OPS) codes, almost perfect autocorrelation sequences (APAS), and zero-correlation-zone sequences (ZCZ) [29,30]. While many of these codes have been investigated for use in phase-coded FMCW (PCFMCW) MIMO radar systems [31,32], the benefits gained from each are shared with phase-coded CW radar. From [18,19], there is clear potential for PMCW radar systems to support low radar-radar interference, provided the phase encoding is unique to each radar in the application space. Moreover, a sufficient knowledge base from decades of communication systems research and implementation, as well as a suitable performance level of commercially available digital components has helped propel the research of digital radar systems. Following years of conceptualization and simulation, PMCW radar systems have started to be explored in depth, mainly through the development of radar systems-on-chip (SoC) with built-in digital modulation [33,34]. While the main industry targeted by these SoC radar systems is automotive applications, event recognition and vital sign detection are also being explored [35,36,37].

Even with the growing interest in exploring PMCW radar systems, few fully realized systems have been demonstrated. In [33], a MIMO PMCW SoC developed by IMEC with 2 transmitters and 2 receivers is detailed along with measurements of stationary corner reflectors. For this device, distance measurements are performed on-chip and transferred to a computer for postprocessing. In [34], a more advanced PMCW MIMO radar SoC developed by Uhnder is detailed, utilizing 12 transmitters and 16 receivers along with internal self-interference cancellation, data acquisition, and on-chip processing. Moving vehicle detection, pedestrian detection, corner reflector response, and digital beamforming sidelobe levels are presented, as well as comparisons to other 77/79 GHz SoCs. In [35], an FMCW radar SoC is compared with a benchtop binary PMCW radar built from high-end evaluation circuits. These systems were used to evaluate current off-the-shelf components for use in automotive radar applications, such as measuring target range, multiple target separation, and moving target response. In [36], various events were classified using a PMCW radar developed by K&G Spectrum Inc. Preliminary results demonstrated the potential for PMCW radar systems for use in identifying stationary subjects and their activities, along with heart rate and respiration measurements. In [37], a PMCW radar response is simulated and compared to the measured response of a PMCW radar prototype also developed by K&G Spectrum Inc. Here, vital sign information was extracted using Time-Frequency Representation (TFR), and possible scenarios were identified using the multiple signal classification (MUSIC) algorithm.

PMCW radar systems have some drawbacks compared to FMCW radar systems, such as additional loads being introduced in the processing chain, the incorporation of digital modulation into the carrier wave, and the Doppler shift intolerance of PRBSs when measuring the target range. Advancements in digital signal processing and field programmable gate arrays (FPGA) can help lighten the processing load. High-speed wideband binary phase shifters (BPS) have also been developed, and PMCW radar integrated circuits (IC) have integrated methods of introducing phase modulation [33,34]. For the Doppler shift intolerance, increasing the length of the PRBS helps to alleviate the loss in the maximum unambiguous range of the target [38]. 

While there are extensive studies on realized FMCW radar systems for vital sign and gesture detection, most real-world PMCW radar measurements have been focused on automotive applications. In this paper, a realized multimode radar system is presented along with a comparison between digitally modulated radar responses of respiration and simple hand gestures. The radar system consists of an RF front-end board that can operate in binary phase-modulated and frequency-modulated CW modes, an oscilloscope for data acquisition, and an FPGA to implement the binary phase modulation of interest. To assess the accuracy of the system over various PMCW modes when measuring breathing cycles, a respiration belt is used as the ground truth (GT). Simplified hand gesture responses are also compared across the various modes. The pros and cons of each mode are discussed, along with the practicability of PMCW radar systems for use in vital sign and gesture detection.

## 2. System Overview

### 2.1. Multimode Digitally Reconfigurable Radar System

**Figure 1:**
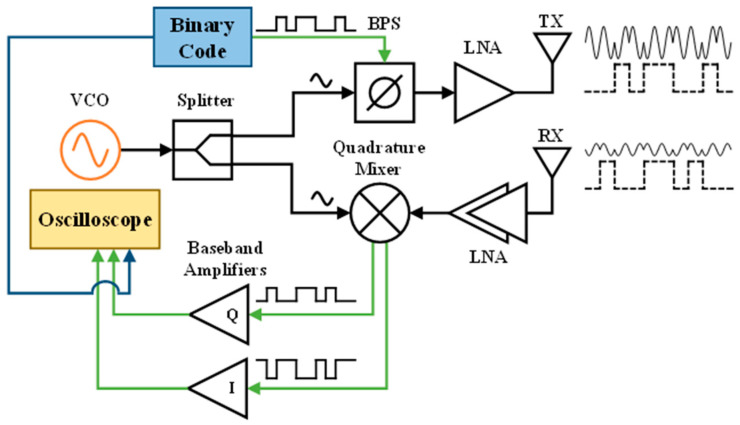
Block diagram of the digitally reconfigurable multimode radar system. VCO: voltage-controlled oscillator; BPS: binary phase shifter; LNA: low noise amplifier. The Binary Code block is implemented through an FPGA that controls the two complimentary BPS inputs and allows for easy switching between phase-modulated operating modes.

To introduce digital modulation schemes to the transmitted waveform and to create the multimode radar, a CW radar system is modified by introducing a wideband BPS in the transmitter chain. The block diagram for the radar system is shown in Figure 1.

The reconfigurable system consists of an external field programmable gate array (FPGA) board to provide the binary phase shifting schemes to the BPS, external voltage-controlled oscillator (VCO), RF front-end board, baseband board, and four-channel oscilloscope acting as the data acquisition device (DAQ). Three of the channels are used to sample the radar system, two for the in-phase (I) and quadrature (Q) signals from the RF front end, and the third for the digital reference signal from the FPGA. The fourth channel is used to measure a ground truth signal from a respiration belt when measuring vital signs.

**Figure 2:**
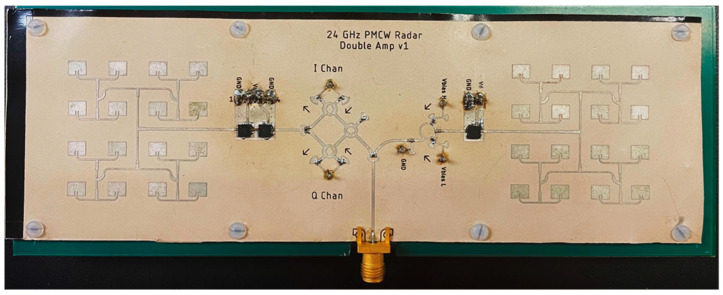
*K*-Band multimode radar RF front-end consisting of TX/RX antennas, the BPS, quadrature mixer, VCO input, and TX/RX LNAs. The 10 mil Rogers 3003 board is mounted to a 63 mil baseband PCB for structural stability.

To realize this system, a planar K-Band RF front-end was designed on 10 mil (0.25 mm) Rogers 3003 substrate. This substrate choice is mainly influenced by the material properties between 8 and 40 GHz, such as the low loss tangent of 0.001 and low dielectric constant of 3.00. These properties combined with the 10 mil thickness allow for a small microwave passive component form factor while designing for modern PCB manufacturing tolerances. The RF front-end, shown in Figure 2, consists of three MACOM MAAL-011111 low-noise amplifiers (LNA), two 4 × 4 linearly polarized patch antenna arrays, Wilkinson power dividers, a wideband binary phase shifter, and a passive balanced quadrature mixer. All the RF components were designed and simulated using Ansys HFSS and AWR Microwave Office.

**Figure 3:**
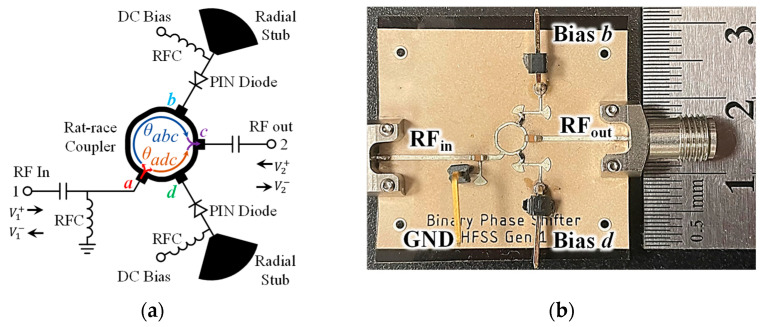
Rat-race coupler-based BPS schematic showing the two possible signal paths from port a to port d along with the reflective loads (**a**) and the fabricated BPS test circuit (**b**) [39].

For the BPS, the design follows [39], which takes advantage of the wideband nature and 180° output phase paths of a rat-race coupler to control the phase delay of the carrier frequency. Rat-race couplers are commonly used to split a signal into two paths with inverted phases while utilizing an isolation port. If instead the two outputs are loaded, as shown in Figure 3a and implemented in Figure 3b, the isolation port becomes a through port with the amplitude and phase of the output controlled by these loads. 

The scattering parameters of the BPS can be defined as functions of the reflective loads $\Gamma_{b}$ and $\Gamma_{d}$ as

(1) $$S_{BPS}=\left[\begin{array}{cc} {S\prime }_{11} & {S\prime }_{12}\\ {S\prime }_{21} & {S\prime }_{22} \end{array}\right]=\frac{-1}{2}\left[\begin{array}{cc} \Gamma_{b}+\Gamma_{d} & {-\Gamma }_{b}+\Gamma_{d}\\ {-\Gamma }_{b}+\Gamma_{d} & \Gamma_{b}+\Gamma_{d} \end{array}\right].$$

To generate the two possible phase states, the b and d inputs to the phase shifter need to have two states they can switch between, and the input loads need to be nearly identical. For best performance, the states need to have high reflection coefficient magnitudes and be 180° out of phase, such as a pair of loads consisting of an RF open and an RF short. By controlling the bias levels of two high-speed PIN diodes with RF short stubs at their anodes, one such state pair is achieved when one diode has a 0-volt bias and the other diode has a forward voltage bias. Flipping the bias states at the same time inverts the phase of the output and achieves the desired binary performance. In this design, MACOM MADP-000907-14020P PIN diodes with switching times of 2 ns were used, allowing the BPS to modulate the carrier signal phase up to 10 GHz if needed. This BPS has an operating bandwidth of 5.72 GHz centered on 24.74 GHz, which covers the radar system’s design bandwidth and has an absolute phase difference greater than 170° between the states across this bandwidth. The insertion loss across this bandwidth is less than 5 dB, and the input matching is less than −15 dB. 

The passive quadrature mixer used in this radar design is adapted from [40]. It consists of two balanced mixers, each made with a 90° hybrid coupler and two Skyworks SMS7621-060 Schottky diodes. The mixer uses the unmodulated carrier split by another 90° hybrid coupler as the local oscillator (LO) input and the modulated RX signal reflected off the target split by a Wilkinson power divider as the RF input. This arrangement mixes the LO and RF to downconvert the RX signal to low-frequency baseband signals that contain only the modulation and target information. This way, the 90° phase difference for the desired I and Q responses for target distance null-point removal is achieved and various I/Q processing methods are possible, such as arctangent or linear demodulation.

The VCO chosen to feed into the SMA connector is an Analog Devices HMC739 operating at a carrier frequency $F_{C}\;$ of 24.125 GHz and has a total frequency range of 23.8 to 26.8 GHz. All passive structures were designed with operating bandwidths of at least 4 GHz centered at $F_{C}$. The 4 × 4 patch antenna array was chosen for the high directivity to reduce multipath and off-axis interferences, as well as the high gain. It is also planar, easy to fabricate, and straightforward to integrate into the radar using microstrip waveguides. The Wilkinson power dividers used for splitting the VCO to the BPS and I/Q channels of the mixer have an S21 and S31 equal power split of −4 dB along with better than −10 dB input matching at all ports across the operating bandwidth. The I/Q channels are fed through identical AC coupled baseband amplifiers and combined to form the complex I/Q response I+jQ.

### 2.2. Digital Modulation Schemes

As stated, this radar system can support a variety of operating modes, all of which will have the BPS input logic follow b=~d. For this paper, the focus is on the PMCW modes through the introduction of digital codes by switching the phase of the carrier between two available phase states. The CW mode is enabled when the BPS inputs are constant. The main use for this mode is to recover unmodulated Doppler frequency responses, such as target motion, respiration, and hand gestures. In CW mode, the radar power consumption, transmitted power, and received power can also be measured and $F_{C}$ can be tuned.

By introducing a repeating binary string of $\left\{01\right\}$ to the BPS, the output waveform becomes a phase-shift keyed (PSK) signal. This mode convolves a square wave onto the carrier frequency $F_{C}$ at the binary modulation frequency $F_{Clk}$ which gets reflected off the subject while encoded. The phase of the introduced PSK wave can be described as

(2) $$\theta_{PSK}\left(t\right)=\theta_{0}+\frac{∆\theta }{2}\times \mathrm{sign}\left(\sin\left(2\pi F_{Clk}t\right)\right)$$

where the phase difference ∆θ is the absolute difference between binary states of the BPS. For the lowest PSK bit error rate using a BPS, the ideal ∆θ should be 180°.

**Figure 4:**
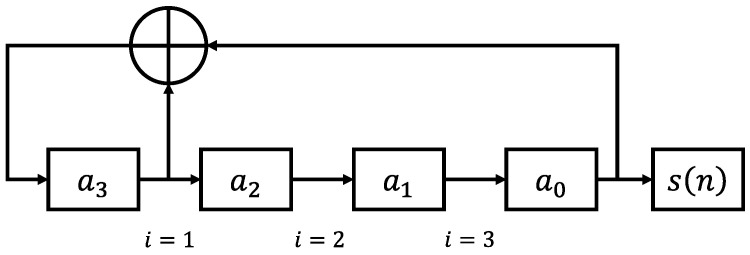
LSFR for generating the MLS described in (5). The cyclic 15-bit binary sequence seen at $a_{0}$ for a starting sequence $\left\{a_{3},a_{2},a_{1},a_{0}\right\}=\left\{0,0,0,1\right\}$ is 100011110101100. This repeating sequence can be expanded by increasing the number of shift registers, as well as by adjusting the location and number of XOR feedback nodes to match higher-order primitive polynomials.

A PRBS is used for PMCW operation, which acts similarly to the PSK mode while having the recovered waveform also contains the binary sequence. This can be used to isolate the recovered signal from other PMCW radar systems operating in the same area and frequency band, be used in joint-radar communication systems, or be a TX/RX channel in a CDMA bistatic MIMO radar system. For this system, an MLS was chosen as the PMCW modulation scheme. These sequences have desirable orthogonality properties, being balanced and having a single point of autocorrelation following

(3) $$\theta_{x,x}\left(0\right)=N\;\mathrm{and}\;\theta_{x,x}\left(l\right)=-1\;\mathrm{for}\;1\leq l<N\;\mathrm{and}\;l=l\;mod\;N,$$

where N is the period of the sequence and l is the cyclic shift amount of the original sequence [41]. There are a finite number of sequences for each degree of m with a length of $N=2^{m}-1$. Each binary MLS also has a polynomial interpretation over GF(2), a finite or Galois field of order 2, which must be a primitive polynomial of degree m. These polynomials take the form

(4) $$P\left(x\right)=x^{m}+a_{m-i}x^{m-i}+\cdots +a_{0}\;\mathrm{for}\;m>i$$

and can be directly converted to the tap arrangement in a linear feedback shift register (LFSR). The sequences can be dynamically generated with a digital LSFR if the registers have any starting value other than all zeros, which will never advance from this initial state as the feedback will always be zero. Alternatively, since the cyclic binary states are deterministic, they can be pre-generated to ensure accuracy and loaded into a memory register on the FPGA, which then increments a pointer through the MLS before looping around to the start of the sequence. An example primitive polynomial of degree 4 is

(5) $$P\left(x\right)=x^{4}+x^{1}+1,$$

its corresponding LSFR arrangement is shown in Figure 4.

The equation for the phase of the PMCW waveform is similar to (2), but the sign of the phase offset is obtained through a piecewise equation that corresponds to the pseudorandom equation of the sequence. For an MLS, the sign can be defined as the equation $s\left(t,m\right)$ with the number of intervals m equaling the order of the primitive polynomial used to make the sequence. Using the MLS described in (5), $s\left(t,m\right)$ can be defined as

(6) $$\begin{array}{c} s\left(t,m=4\right)={2a}_{0}-1\;\mathrm{where}\left\{\begin{array}{cc} a_{3}\left[n+1\right]= & mod\left(a_{0}\left[n\right]+a_{3}\left[n\right],2\right)\\ a_{2}\left[n+1\right]= & a_{3}\left[n\right]\\ a_{1}\left[n+1\right]= & a_{2}\left[n\right]\\ a_{0}\left[n+1\right]= & a_{1}\left[n\right] \end{array}\right\}\\ \mathrm{for}\;n=mod\left(\left\lfloor F_{Clk}\times \left(t\right)\right\rfloor +n_{0},2^{m}-1\right)\mathrm{and}\mathrm{nonzero}\mathrm{initial}\mathrm{conditions}, \end{array}$$

where $n_{0}$ is the initial bit of the MLS and $F_{Clk}$ is the clock speed used to cycle through states. The generated sequence for this MLS with initial conditions $a_{0}\left[0\right]=1,\;a_{1,2,3}\left[0\right]=0$ is given in Table 1.

The added phase to the PMCW carrier wave is then

(7) $$\theta_{PMCW}\left(t\right)=\theta_{0}+\frac{∆\theta }{2}\times s\left(t,m=4\right),$$

with the lowest PMCW symbol error rate using a BPS occurring when ∆θ=180°.

### 2.3. Target Response Recovery and Demodulation

Assuming the TX signal from the radar in CW mode is

(8) $$TX\left(t\right)=A_{\mathrm{TX}}\cos\left(2\pi F_{C}t+\theta_{TX}\right),$$

it follows that by modulating the phase of the carrier with $\theta_{m}\left(t\right)$, which includes the initial phase constant $\theta_{TX}$, this equation can be modified to

(9) $$TX\left(t\right)=A_{\mathrm{TX}}\cos\left(2\pi F_{C}t+\theta_{m}\left(t\right)\right).$$

The target vital sign or gesture information is then mixed with the carrier signal as it reflects off the target, which is then downconverted in the quadrature mixer and measured at the output. The RX signal is downconverted with the unmodulated carrier $\cos\left(2\pi F_{C}x\left(t\right)+\theta_{0}\right)$, and the initial phase constants can be combined into a DC component. $\theta_{m}\left(t\right)$ is then replaced by (2) in the PSK mode or (7) in the PMCW mode.

Since this radar design uses a quadrature mixer, the outputs of the radar to the DAQ are the desired I and Q signals. As adapted from [25], the CW, PSK, and PMCW I/Q baseband responses can be defined as:

(10) $$B_{{CW}_{I}}\left(t\right)=A_{I}\cos\left(4\pi x\left(t\right)/\lambda_{C}+∆\varphi \left(t\right)\right)+{DC}_{I}$$

(11) $$B_{{CW}_{Q}}\left(t\right)=A_{Q}\cos\left(4\pi x\left(t\right)/\lambda_{C}+∆\varphi \left(t\right)\right)+{DC}_{Q}$$

(12) $$B_{{PSK}_{I}}\left(t\right)=A_{I}\cos\left(4\pi x\left(t\right)/\lambda_{C}+∆\varphi \left(t\right)+\theta_{PSK}\left(t\right)\right)+{DC}_{I}$$

(13) $$B_{{PSK}_{Q}}\left(t\right)=A_{Q}\cos\left(4\pi x\left(t\right)/\lambda_{C}+∆\varphi \left(t\right)+\theta_{PSK}\left(t\right)\right)+{DC}_{Q}$$

(14) $$B_{{PMCW}_{I}}\left(t\right)=A_{I}\cos\left(4\pi x\left(t\right)/\lambda_{C}+∆\varphi \left(t\right)+\theta_{PMCW}\left(t\right)\right)+{DC}_{I}$$

(15) $$B_{{PMCW}_{Q}}\left(t\right)=A_{Q}\cos\left(4\pi x\left(t\right)/\lambda_{C}+∆\varphi \left(t\right)+\theta_{PMCW}\left(t\right)\right)+{DC}_{Q}$$

In these equations, $x\left(t\right)$ is the phase shift imparted into the reflected waveform by the target’s motion relative to the radar. $\lambda_{C}$ is the wavelength of the carrier frequency, $∆\varphi \left(t\right)$ is the noise in the environment, $A_{I}/A_{Q}$ are the AC amplitude of the signal out of the mixer, and ${DC}_{I}/{DC}_{Q}$ are the DC offset of the I/Q channels, respectively. As the modulation schemes used by the FPGA are cyclic and known, the main challenge faced in baseband processing is the FPGA clock and the oscilloscope clock being asynchronous. To address this, the modulation scheme $s_{in}(t)$, as well as the I/Q channel data, is measured with the oscilloscope sampling well over twice of $F_{Clk}$. An oversampling factor greater than two is needed to satisfy the Nyquist sampling theorem and recover at least one point per modulation symbol for accurate demodulation. Therefore, to ensure high-resolution sampling of the asynchronous modulation signal and to account for clock speed imprecision, a sampling rate of ${50\times F}_{Clk}$ was chosen. With this information, various approaches can be taken to demodulate the PMCW baseband I/Q signals that can also be extended to the PSK baseband signals. 

For this radar system, all the signal processing was performed in MATLAB. PMCW radar systems when used for measuring distance have a range resolution of $c/\left(2\times F_{Clk}\right)$, as the phase difference between the transmitted and received code will have transitioned one or more encoded symbols over the signal’s travel time. For these experiments, $F_{Samp}$ and $F_{Clk}$ are chosen to be much lower than the frequency needed to accurately measure phase differences between the transmitted and received signals, so target distance cannot be determined. There will still be a time offset between the recovered I/Q basebands and $s_{in}$ due to differences in the signal processing chains, in which I/Q are AC coupled and amplified, so a small offset is applied to align the logical transition edges of measured signals, which will not be separated by more than $1/F_{Clk}$. After alignment, the transition edges of $s_{in}$ are located by taking its first derivative, and the transition type of low–high or high–low is determined with its second derivative. With these edges, the number of samples in the shortest measured symbol is found, across which in an MLS symbol will span one clock cycle and in PSK will span a half clock cycle. From here, the binary sequence is extracted and $s_{in}$ is partitioned into $F_{Samp}/\left({2\times F}_{Clk}\right)$ sections where the sign of the section is determined.

After aligning the transition edges in $s_{in}$, the modulated I/Q signals are separated into logical low and high portions. Each of the windows obtained from processing $s_{in}$ are applied to the time and AC coupled I/Q signals, in which each window is averaged. The logical highs and lows in $s_{in}$ are used to rectify and merge the corresponding halves of the I/Q signals. It is important to note that every MLS will have an odd number of symbols, resulting in the sum of the high and low logic states being off by one. Therefore, to correct the DC offset generated by the extra symbol, each of the logical low and high portions of the I/Q signals are shifted toward each other. Then, the low and high I/Q signals are interpolated and resampled at a rate of $F_{Clk}$. This allows them to be aligned with the windowed and averaged time information, and each pair of low and high channel data are averaged to get the target motion I/Q responses.

## 3. Measurement Results

### 3.1. Transmitted Phase Modulated Power Spectrums

While the output of CW radar has a constant average and instantaneous power, once modulation is added the carrier power spreads out over the frequency spectrum. For FMCW radar systems, the carrier frequency is constantly moving across the operating spectrum. This is commonly implemented by applying a periodic sawtooth ramp to the tuning voltage of the VCO, which ideally results in a constant change in the carrier frequency until resetting from the highest frequency to the lowest. As the frequency sweeps across the operation bandwidth, the output power should be constant. For PMCW radar systems, the instantaneous power across phase states should also not change, while the average power follows the equation

(16) $$PS\left(f\right)=F_{C}\left(f\right)*F_{M}\left(f\right)$$

where $F_{C}\left(f\right)$ and $F_{M}\left(f\right)$ are the frequency components of the carrier and the modulated phase, respectively.

**Figure 5:**
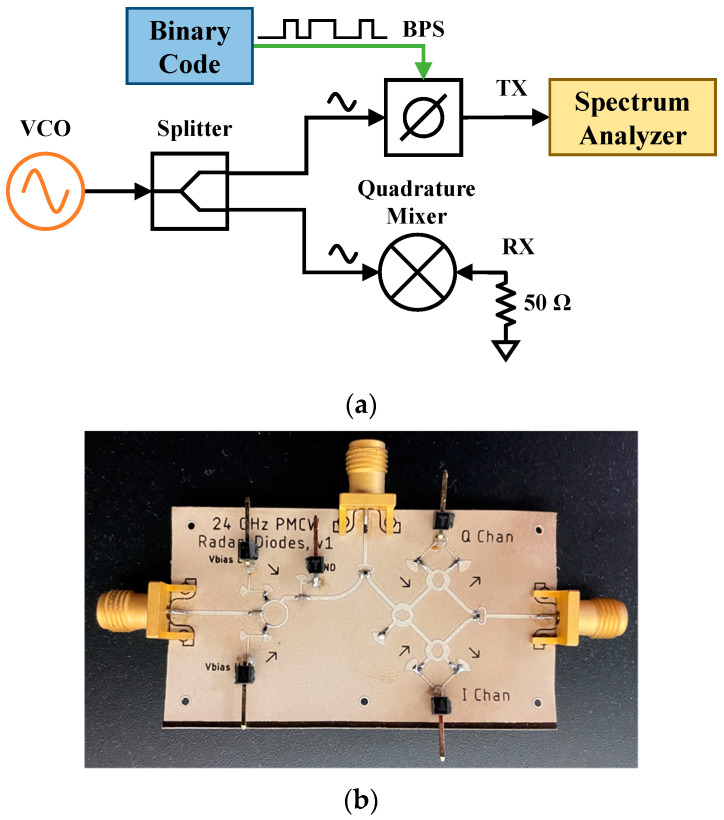
RF front-end test circuit (**a**) block diagram and (**b**) corresponding test board. For spectral analysis, the VCO is connected to a variable frequency generator, the output of the BPS is measured with a spectrum analyzer, and the RF input to the mixer is loaded with 50 Ω.

**Figure 6:**
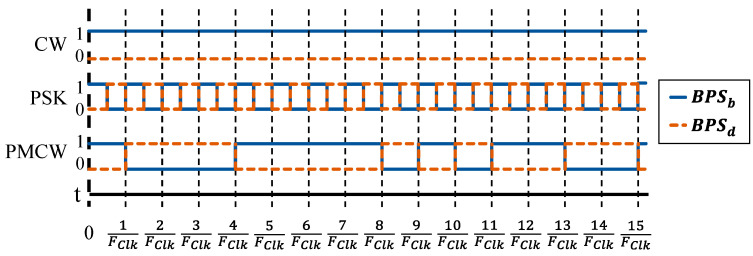
Logical timing diagram for the BPS bias voltages corresponding to the radar modes in Table 2. The PSK signal repeats at the frequency $F_{Clk}$ and the PMCW symbol updates on every rising clock edge.

To ensure the RF front-end of the multimode radar is properly modulating the carrier signal, a test board with the block diagram in Figure 5a was developed. The fabricated test board is shown in Figure 5b. Three possible output modes of the RF front-end were tested using the binary input schemes in Table 2 and a logical timing diagram for the phase states of each waveform is shown in Figure 6.

**Figure 7:**
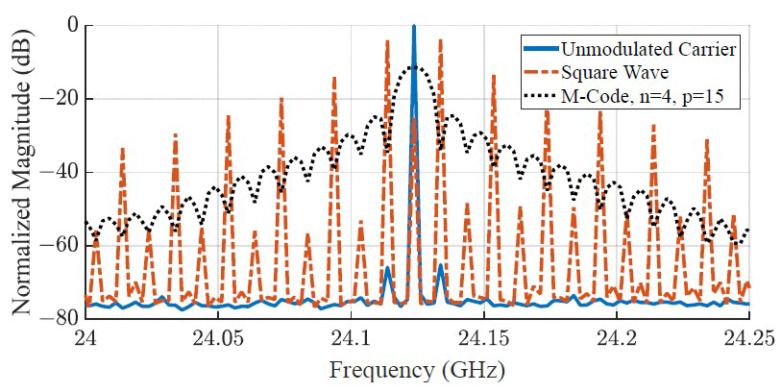
Measured frequency spectrums that compare the average output power of an unmodulated carrier tone at 24.125 GHz to PSK and PMCW carrier waves running at an $F_{Clk}$ of 10 MHz. As the carrier modulation becomes less periodic, the power spreads out over the spectrum.

When applying PSK and PMCW modulation schemes to the BPS, the power of the output wave is spread across the spectrum, resulting in lower average power compared to the unmodulated carrier at $F_{C}$. To compare the average output power to an FMCW radar, the VCO frequency is swept while the BPS states in the front-end test board are in CW mode. The average power spectrum of the FMCW wave is also spread out over time depending on the chirp rate $F_{Chirp}$. Since both MLS-based PMCW and FMCW carriers are periodic, the average power spectrum per cycle is compared. Figure 7 shows the output spectrum of phase shifting schemes. The $F_{Clk}$ used for the comparisons in Figure 7 is 10 MHz for both the PSK square wave modulation and the 15-bit MLS encoding, which repeats every 1.5 µsec. 

**Figure 8:**
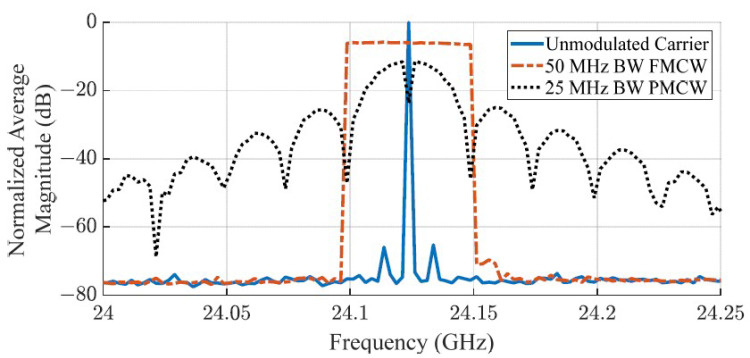
Comparison of average output power per cycle for CW, FMCW, and PMCW waveforms measured with the RF front-end test circuit. The bandwidth occupied by the FMCW waveform is similar to that of the PMCW waveform’s main lobe when the carrier’s phase is modulated with a clock speed that is half of the swept FMCW bandwidth.

Figure 8 shows the normalized output power spectrum per cycle of CW, FMCW, and PMCW signals. As the main lobe of PMCW waveforms occupies $2\times F_{Clk}$, the available bandwidth is half of that which is accessible by FMCW waveforms. Figure 8 demonstrates this concept with a 50 MHz bandwidth FMCW spectrum that uses a 1 kHz sawtooth wave to sweep the carrier frequency and a 15-bit MLS-PMCW spectrum with a 25 MHz clock frequency, repeating every 0.6 µsec. The FMCW signal will have twice the range resolution of the PMCW signal because of this drawback but will also suffer from high radar–radar noise when operating in the same band as another FMCW radar.

### 3.2. Radar Sensitivity

**Figure 9:**
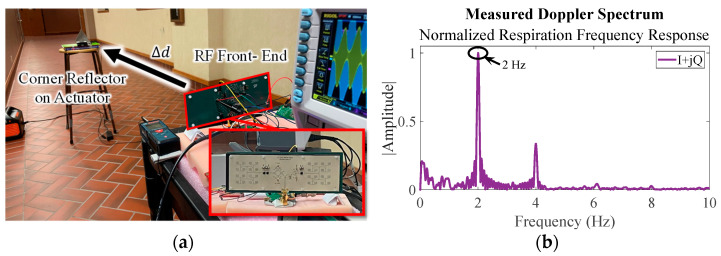
Sensitivity test measurement setup (**a**) and baseline Doppler frequency response for a 1 m target with 1 mm motion at 2 Hz (**b**). The distance, oscillation frequency, and oscillation motion were isolated and taken to their respective response limits to determine the Doppler sensitivity.

To test the Doppler sensitivity of the reconfigurable radar, a corner reflector was positioned in front of the system, as shown in Figure 9a. The motion amplitude, motion frequency, and distance to the radar were isolated and individually swept. The initial test setup used was 1 mm motion at 2 Hz and 1 m away, and its I/Q response is shown in Figure 9b. From here, the variables of interest were swept until the spectral response did not contain the motion frequency, and the limits of the system are given in Table 3.

### 3.3. Respiration Detection

**Figure 10:**
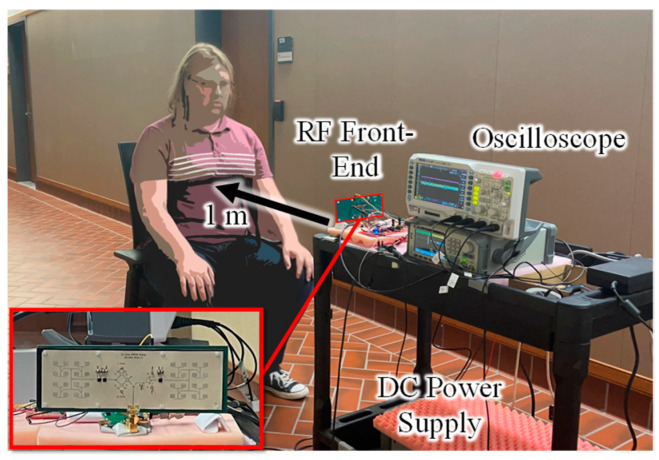
Respiration measurement setup. Target’s chest is 1 m away from the radar system.

In Figure 10, the multimode radar system is used to measure the respiration of a target using CW, PSK, and PMCW waveforms. For this setup, the same three modes measured in Section 3.1 are used with $F_{Clk}$ at 1 kHz, while the oscilloscope records 60 s of data. The limiting factor in this measurement setup is the memory depth of the oscilloscope, which is 3 million points. Across a measurement window of 60 s, this allows for a sampling rate of 50 ksps and a maximum of 50 points per encoded symbol. The four measurements recorded in each setup are the I/Q signals from the output of the mixer, the modulation scheme from the FPGA to the BPS, and the output of the respiration belt to act as the GT. As human respiration responses most commonly fall between 0.2 Hz and 2 Hz, this longer time window of 60 s was chosen to ensure more than 10 breathing cycles are captured and analyzed.

**Figure 11:**
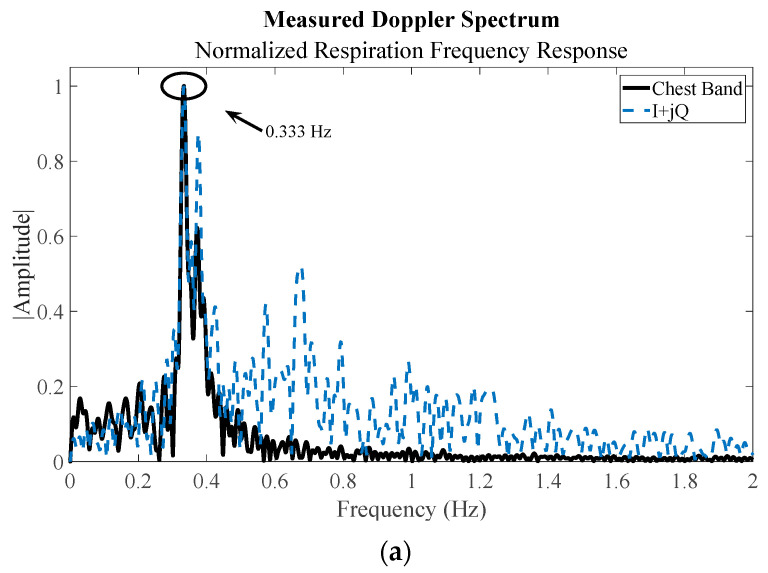

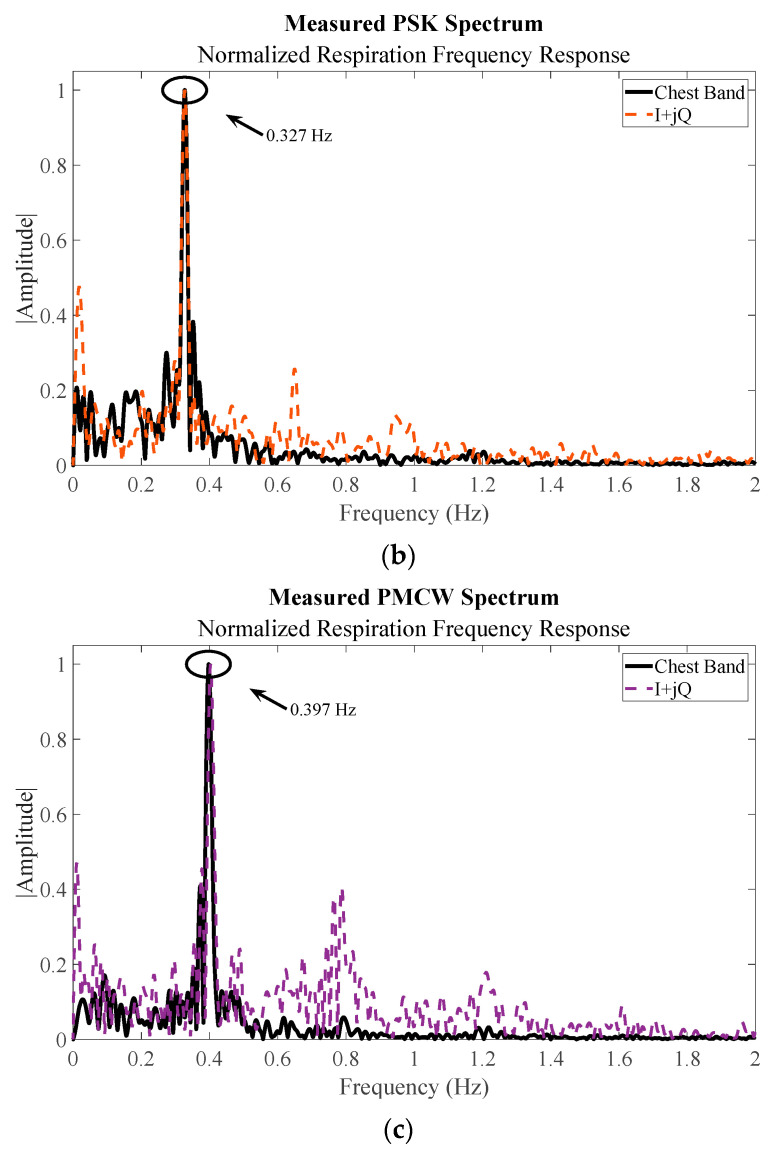
Measured respiration response for (**a**) CW mode, (**b**) PSK mode, and (**c**) PMCW mode. All tests were performed with the subject 1 m from the radar and show a strong correlation with the respiration belt response.

From the equations in Section 2.3, the baseband response of the CW data contains the respiration information, while the baseband PSK and PMCW data need to be decoded following the algorithm outlined in Section 2.2 to recover the low-frequency information. As stated previously, the 50 ksps of the oscilloscope is not fast enough to measure the phase delay of the carrier from the target for PMCW range measurements but is well above the Nyquist sampling rate needed for the 1 kHz modulation rate used. The 1 kHz clock is also significantly higher than any potential frequency information of interest. The measured respiration rates for each mode are compared to their GTs in Figure 11.

### 3.4. Gesture Detection

**Figure 12:**
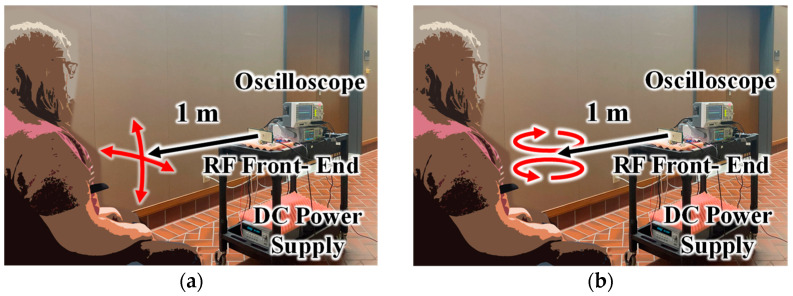
Gesture measurement setup for (**a**) up/down and left/right motion and (**b**) clockwise and counterclockwise rotation. Target’s hand motions are approximately one meter away from the radar system for all tests.

To measure four hand gestures consisting of an up/down motion, left/right motion, clockwise rotation, and counterclockwise rotation, the subject sat around 1.2 m away from the radar with their hand 1 m from the radar, as shown in Figure 12. Over 24 s, each gesture was first repeated three times after waiting four seconds between gestures. For the last gestures in each data set, the translation motion in Figure 12a was performed once at a time, consisting of up-hold-down-hold and left-hold-right-hold patterns, while the rotation motion in Figure 12b was increased to two rotations. The horizontal and vertical gesture motions spanned approximately 0.5 m, and the rotational gestures started 1 m away before rotating toward the radar with a diameter of about 0.3 m. Due to the shorter time window, the modulation signal and I/Q baseband signals of the gestures were recorded at 125 ksps as the oscilloscope could support the same amount of data over a shorter period.

**Figure 13:**
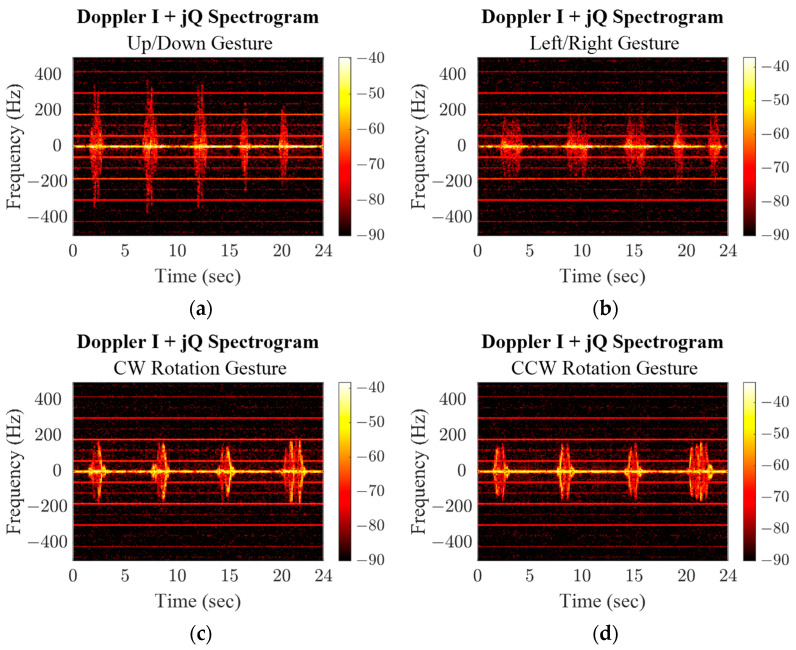
CW mode spectrograms for repeated hand gestures.

**Figure 14:**
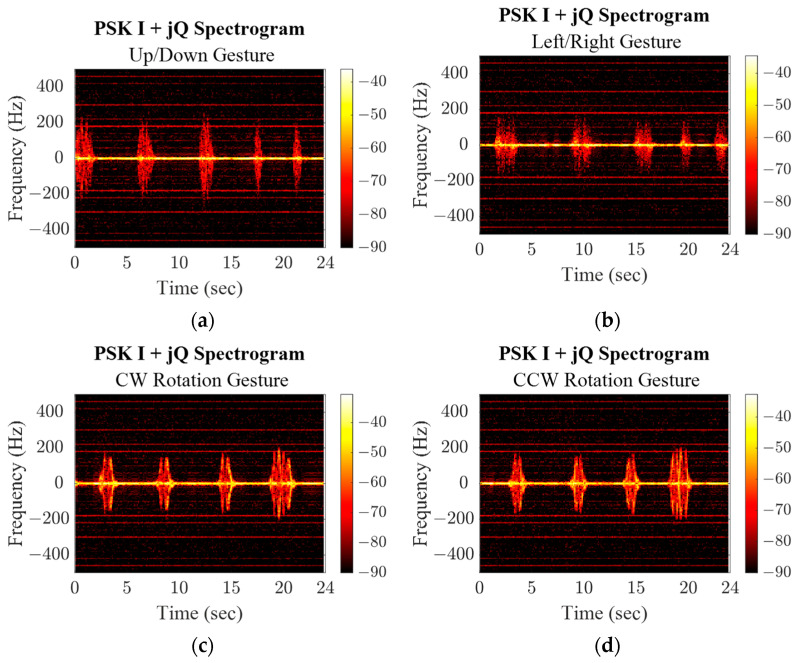
PSK mode spectrograms for repeated hand gestures.

**Figure 15:**
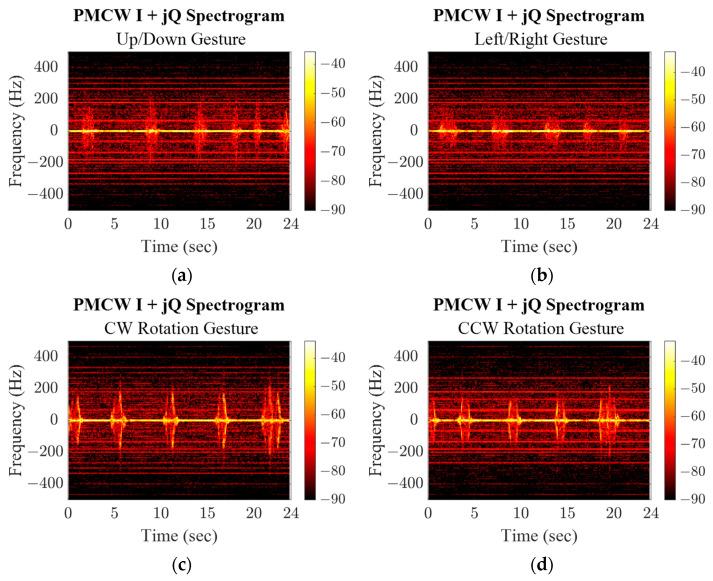
PMCW mode spectrograms for repeated hand gestures.

The PSK and PMCW baseband signals were demodulated, AC coupled, and joined to form a complex response. A set of discrete fast Fourier transforms (DFFT) was then taken from the data with a 1% data window and 50% overlap between windows along the response data for all three radar modes and all four gestures, shown in Figure 13, Figure 14 and Figure 15. The responses of each gesture share identifying characteristics in the amplitude and frequency value of their spectrogram responses. While the up/down and left/right gestures are similar motions and span similar distances, the induced Doppler frequencies are slightly different due to the speed of the gesture. Both straight-line motions are distinctly different from the rotational gesture responses. For the latter, the subject’s location was constant, so as their hand rotated there is a period when their hand reflected more of the transmitted wave and the induced signal is stronger. Since their hand passes through the same space in front of the radar, the timing of the increase in power/frequency is flipped when the rotation is reversed.

## 4. Discussion

While CW and FMCW radar systems have been investigated and experimentally verified for use in noncontact subject monitoring for many years [42,43], demonstrations of the viability of PMCW radar systems in this application space are still sparse. Many studies on PMCW response simulations have been conducted, but the number of realized systems with measured results is low [35]. The multimode radar proposed in this article aims to further research into PMCW subject monitoring and system-level implementation. One challenge faced with PMCW radar measurements is DAQ sampling speed. Without a synchronous digital clock, the radar response and modulation signals need to be oversampled for proper demodulation. A solution to this issue is seen in PMCW radar SoCs, which circumvent this issue by using the same clock for sampling and modulation. For the 1 kHz clock used here, oversampling is achievable using commercial DAQs or oscilloscopes, but this clock speed does not allow for distance measurements through phase delay analysis that is achievable with higher time resolution analysis.

Of the tested waveforms, the CW mode is the simplest to implement and measure. Relying only on the Doppler response, target motion is recovered from the baseband signals without needing any additional demodulation or requirements on sampling frequency outside of being high enough to measure the imparted frequency. It is also the most limited mode, since there is no additional information that can be gained from the response without introducing modulation. The PSK response is slightly more flexible in how it can be processed, as it does not require complex demodulation approaches to extract low-frequency information that is modulated by a square wave. It also upshifts the frequency response, meaning DAQs with low cutoff frequencies would still be able to acquire the Doppler response centered around $F_{Clk}$. Target distance can also be estimated by measuring the phase delay of the transmitted and received phases, but the unambiguous range is limited to one range bin due to the repetition length of the encoding. PMCW modulation, however, is far more flexible for determining range and capable of generating orthogonal signals. As mentioned, various binary phase encodings have been studied for their beneficial autocorrelation properties. These allow for unambiguous ranging that is dependent on code length, while range resolution is dependent on $F_{Clk}$. These benefits require more processing and measurement time to recover and demodulate the PMCW response, and there are limited short PSRB codes. Despite this, as shown in the measurement results, the impact is low enough that respiration responses and gestures can still be recovered.

Low-frequency noise also impacts the measurements. The main constant amplitude frequencies seen in the CW mode spectrogram response are noise from the 60 Hz power grid and its harmonics. For the PSK and PMCW modes, since the carrier is a mix of $F_{C}$ and the modulation wave, the baseband signal is upconverted above this noise level. This frequency shift can be beneficial in many stages of the processing chain, such as in raising the cutoff frequency of a high-pass coupling filter or the improved linearity of baseband amplifiers for higher-frequency signals. This also allows for a variety of DAQ inputs such as computer audio cards, which usually have a cutoff frequency of 20 Hz, to be used for their high resolution and frequency analog-to-digital conversion. The PSK data shows a reduction in background noise due to most of the power being upconverted to the first harmonic of the square wave at $F_{Clk}$ and then taking a windowed average of the response. The PMCW data ends up with an overall raised noise floor as any noise is spread across the frequency spectrum as opposed to being upconverted to square wave harmonics, but a more directive antenna or a higher power VCO would improve the spectrogram measurements.

Much like the current direction of FMCW radar systems for vital sign detection, this system is contactless which allows for more flexible subject monitoring while maintaining measurement accuracy, and the design concepts can be incorporated at higher frequency bands. Moreover, the radar demonstrates that respiration measurement accuracy is not lost when implementing phase modulation, allowing for multiple PMCW radar systems to share the same frequency space, additional information to be encoded onto the phase modulation, and expansion into a monostatic MIMO system. For each of the three radar modes, CW, PSK, and PMCW, the demodulated I/Q response peak frequency strongly aligns with the corresponding peak frequency of the respiration belt over the 60 s measurement window. Here, the subject’s breathing was voluntary and controlled, but respiration rates will vary depending on factors such as but not limited to stress levels, mood, and current health [25]. Speaking also causes breathing to be abnormal. However, these variations will affect the frequency spectrums of the measurements from both the respiration belt and radar systems equally, regardless of the modulation method used.

Measuring hand gestures has also been explored with FMCW radar systems [7,8,9], extended to UWB radar systems [22], expanded to sign language recognition using UWB radar systems [23], and demonstrated using passive radar systems [17]. PMCW gesture and event recognition, however, are still emerging [36]. The spectrogram responses of the four hand gestures measured here show that the low-frequency Doppler information is maintained across both PSK and MLS-based PMCW radar modes. This is encouraging as the low-frequency modulation used in this work can be expanded to CDMA-MIMO radar systems, or joint-radar communication systems, which look to introduce additional information to the carrier wave. Both would allow for more complex clinical research through encoding the radar with identifying patient information, multipatient monitoring, and 3D patient vital sign and gesture measurements.

The radar system detailed here also allows for future improvements in FPGA and DAQ systems to be incorporated. For this design, the microwave structures can be implemented for higher carrier frequencies, and the BPS reflective loads can be modified to allow for faster phase switching. The BPS inputs can be controlled with faster FPGAs as the technology improves, and the baseband response measurements and processing can be moved onto the FPGA to accommodate single-bit PMCW processing used by the PMCW SoC radar systems. 

## 5. Conclusions

As the applications and demands of modern radar systems grow, current and future frequency spectrum coexistence needs to be considered. The multimode radar design and approach to implementing digitally reconfigurable PMCW radar signals described in this article demonstrate how a benchtop PMCW radar can be easily designed and reconfigured to test various CW modulation schemes. The experimental results show that while there is an increased load on the baseband processing, vital sign and simple gesture detection is achievable with both PSK and PMCW radar systems. Four gestures were measured that have identifying characteristics between one another, and respiration measurements strongly correlate with those measured with a respiration belt. Future works will focus on identifying and classifying a wider array of subjects’ gestures and movements, as well as an in-depth comparison to CW and FMCW responses. Further exploration of PMCW respiration responses will investigate scenarios with more subjects at various distances, as well as multiradar and multipatient scenarios, heart rate detection, and supporting passive radar systems.

## Figures and Tables

**Table 1:** LSFR sequence for (5), which repeats every 15 bits. This repeating binary sequence is translated to each of the two possible phase states generated by the BPS, where the logical 0 state is chosen as the in-phase state and the logical 1 as the 180° out-of-phase state. With the use of the FPGA’s digital output signals, the differential pair of low and high voltage levels modulate the phase of the carrier from the VCO.

	n	0	1	2	3	4	5	6	7	8	9	10	11	12	13	14
$a_{i}$																
$a_{3}$		0	1	1	1	1	0	1	0	1	1	0	0	1	0	0
$a_{2}$		0	0	1	1	1	1	0	1	0	1	1	0	0	1	0
$a_{1}$		0	0	0	1	1	1	1	0	1	0	1	1	0	0	1
$a_{0}$		1	0	0	0	1	1	1	1	0	1	0	1	1	0	0

**Table 2:** BPS Bias Logic Input Sequences. Each pair of inputs corresponds to the repeating normalized magnitude of the bias voltages supplied by the FPGA.

Radar Mode	${Vbias}_{b}$	${Vbias}_{d}$
CW	$\left\{0\right\}$	$\left\{1\right\}$
PSK	$\left\{01\right\}$	$\left\{10\right\}$
PMCW	$\left\{MLS\right\}$	$\left\{\sim MLS\right\}$

**Table 3:** Radar Doppler motion Sensitivity Parameters.

Max Distance	4 m	@ 1 mm motion, 2 Hz
Min Amplitude	50 µm	@ 1 m distance, 2 Hz
Min Frequency	0.05 Hz	@ 1 mm motion, 1 m distance

## Data Availability

The data presented in this study are openly available in FigShare at DOI: 10.6084/m9.figshare.24082266 and available on request from the corresponding author.
